# Fabrication of Photothermo-Responsive Drug-Loaded Nanogel for Synergetic Cancer Therapy

**DOI:** 10.3390/polym10101098

**Published:** 2018-10-04

**Authors:** Ray Chang, Wei-Bor Tsai

**Affiliations:** 1 Department of Chemical Engineering, National Taiwan University, Taipei 10617, Taiwan; r02524024@ntu.edu.tw; 2 Advanced Research Center for Green Materials Science and Technology, National Taiwan University, Taipei 10617, Taiwan

**Keywords:** nanogel, photothermo-responsive, *N*-isopropylacrylamide, sodium copper chlorophyllin, drug delivery, anti-cancer

## Abstract

Temperature stimulus, easy modulation in comparison to other environmental stimuli, makes thermo-responsive nanocarriers popular in the applications of controlled drug release for cancer therapy. In this study, photosensitive sodium copper chlorophyllin (SCC) was incorporated into thermo-responsive polymeric nanogels consisted of *N*-isopropylacrylamide and *N*-(hydroxymethyl)acrylamide. Significant heat was generated from the SCC-containing nanogels under the exposure to 532-nm green laser, and resulted in cell mortality. The thermo-responsive nanogel loaded with 5-FU, an anti-cancer drug, released the drug explosively when exposed to green laser. The combination of hyperthermia and temperature-induced drug release via green laser irradiation greatly enhanced cell mortality to a maximal extent. Such photothermo-responsive nanogel possesses a great potential in anti-cancer treatment.

## 1. Introduction

Cancer, including a range of diseases caused by the unregulated growth of malignant cells, is one of the major death causes worldwide [1]. Traditional anti-cancer treatments include surgery, radiation therapy, and chemotherapy or a combination of these treatments. Chemotherapy often applies chemotherapeutic agents that interfere cellular DNA replication, leading to the death of fast-duplicating cancerous cells [2]. Even though traditional chemotherapeutic agents show success to some extent, the drugs do not specifically target cancer cells and may damage healthy normal tissue, causing severe uncomfortable side effects, such as mouth sores, diarrhea and vomiting [3]. Recently, the development of nanotechnology provides an alternative solution to overcome these problems. 

Environmentally responsive nanogels are polymeric nanoparticles that respond to environmental changes with alteration of the architecture of the polymer network. Such nanogels are designed for delivering chemotherapeutic agents to tumor sites and unloading the drug suddenly upon environmental changes. This controlled way of drug release potentially minimizes the adverse effects while improving therapeutic efficacy. Therefore, environmentally responsive nanogels have been utilized widely as drug delivery systems for on-demand drug release in recent years [4]. Many types of stimuli, including changes in temperature, pH and electromagnetic field, have been the elements for the design of stimulus-responsive nanogels. Among the stimuli, temperature change has been frequently applied to controlled drug release due to its suitability for external control. Thus, much research attention has been focused on the fabrication of thermo-responsive nanogels [5]. 

Poly(*N*-isopropylacrylamide) (pNIPAAm) exhibits a reversible hydrophobic/hydrophilic transition at its lower critical solution temperature (LCST), ~32 °C. When the surrounding temperature is below LCST, the amido groups on the polymer form hydrogen bonding with water molecules, leading to swelling of the polymer network. On the other hand, when the temperature is raised above LCST, the hydrogen bonds are replaced by intermolecular forces between pNIPAAm chains, resulting in an aggregated polymer network [6]. With the hydrophobicity-transition property, pNIPAAm have been widely used in the fabrication of thermo-responsive nanogels for drug delivery [7]. 

Photothermal agents can be incorporated into pNIPAAm-based nanogels in order to trigger their thermo-response under light illumination [8,9]. Photothermal agents transform absorbed light into emitted heat, which causes local hyperthermia around the nanogels. It has been reported that the anti-cancer efficiency of hyperthermia induced by photothermal agents is higher than traditional hyperthermia methods such as hot-water bath or heated blood perfusion [10]. Moreover, light-induced heat can be used to stimulate rapid drug release from nanogels at a targeted location. 

Most current research uses near-infrared (NIR) light as an external trigger due to its deeper tissue penetration in comparison to visible light [11,12]. However, visible light provides some advantages in certain medical procedures [13]. For example, green light has been applied in the clinical treatment of prostate benign prostate hyperplasia [14]. Although the penetration depth of green light into a tissue is less than NIR [15], the absorption coefficient of green light to water is much lower, nearly 10-fold, than that of NIR [16]. The selective absorption property may make green light suitable for photothermal treatment without heating surrounding tissues. Previously, our group fabricated a nanogel composed of sodium copper chlorophyllin (SCC), a photothermal agent absorbing green light, and *N*-[3-(dimethylamino) propyl] methacrylamide (NDPMA), a positively charged monomer [17]. After being taken up by the cells through electrostatic interactions, the cell-killing efficacy of the SCC-containing nanogel was greatly enhanced via photothermal effect.

**Scheme 1:**
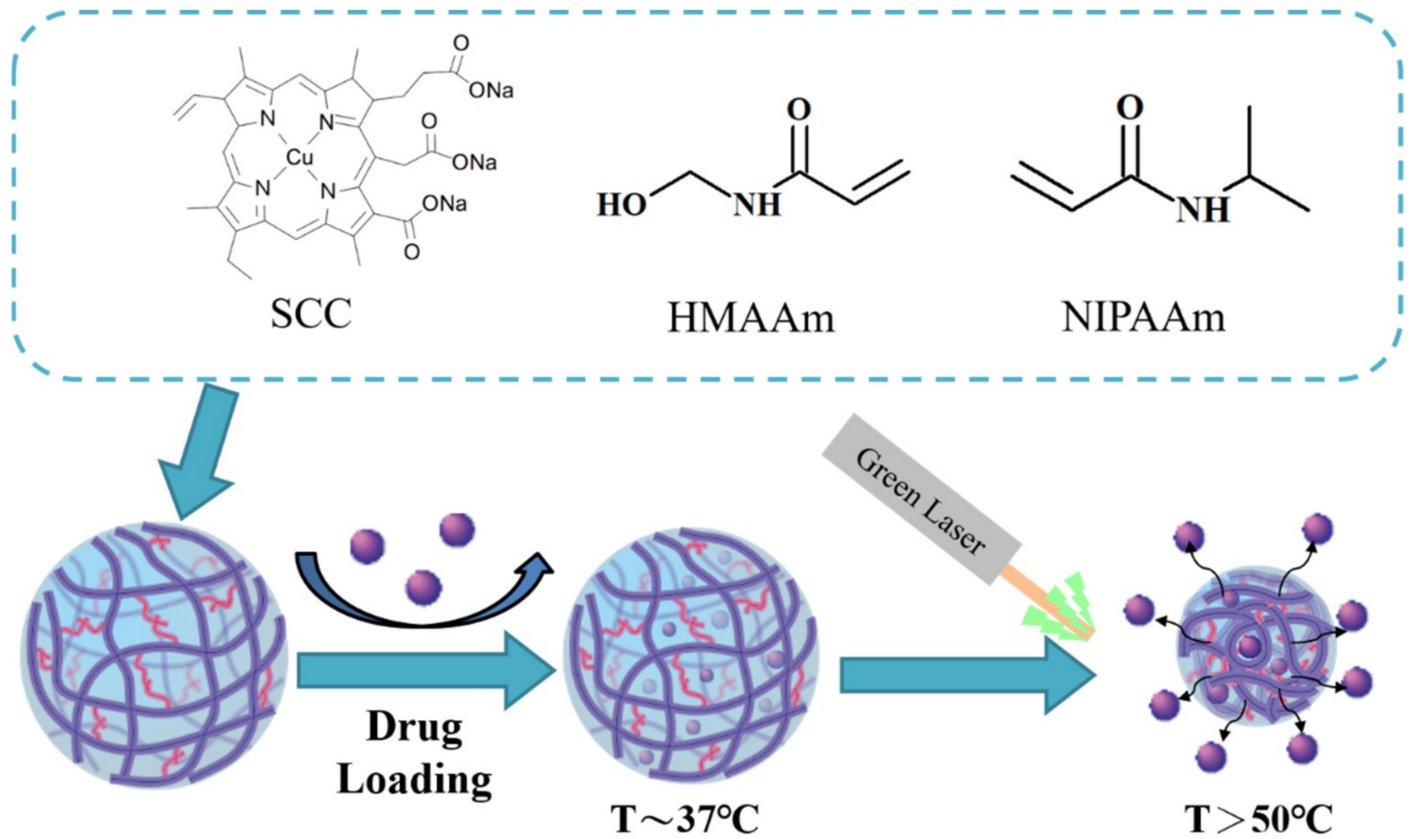
Illustration of drug loading to and green laser-triggered release from pNIPAAm-*co*-HMAAm-*co*-SCC nanogels.

To increase the therapeutic efficiency, in this study, SCC was incorporated into pNIPAAm-based nanogels for the purposes of both photothermal therapy and thermally controlled drug release. *N*-(hydroxymethyl)acrylamide (NHMAAm) was co-polymerized into pNIPAAm nanogels for modulation of LCST in order to adjust responsive temperature. NHMAAm, a hydrophilic monomer, increases the hydrophilic content of the copolymer, and thus increases the LCSTs [18]. The drug-incorporated pNIPAAm-*co*-NHMAAm nanogel would release the drug at a temperature higher than the normal LCST of pNIPAAm. The nanogels could be heated to a temperature (>50 °C) above the normal body temperature on the exposure to green laser and then suddenly release an anti-cancer drug (Scheme 1). Both the elevated temperature and the release drug could kill cancer cells, potentially resulting in a synergic anti-cancer effect. 

## 2. Materials and Methods

### 2.1. Chemicals

*N*-isopropylacrylamide (NIPAAm, cat. no. 415324), *N*-(hydroxymethyl) acrylamide, (NHMAAm, cat. no. 245801), *N*,*N*’-methylenebisacrylamide (MBAA, cat. no. M7279), chlorophyllin sodium copper sodium salt (SCC, cat. no. C6003), 4,4’-azobis(4-cyanovaleric acid) (ACVA, cat. no.11590), thiazolyl blue tetrazolium bromide (MTT, cat. no. M5655), 5-fluorouracil (5-Fu, cat. no. F6627), fetal bovine serum (FBS, cat. no. T8153), 2-mercaptoehanol (cat. no. M3148) and trypan blue (cat. no. T8154) were purchased from Sigma-Aldrich (St. Louis, MO, USA). Sodium dodecyl sulfate (SDS, cat. no. 230421000) was purchased from Acros organics (Bridgewater, NJ, USA). Gentamicin (cat. no. 15710-064) was purchased from Thermo Fisher Scientific (cat. no. F6627; Waltham, MA, USA). L929 cells were obtained from the Food Industry Research and Development Institute (Hsinchu, Taiwan) and maintained in α-MEM (cat. no. 11900-024, Gibco, Waltham, MA, USA) supplemented with 0.5% gentamicin, 0.04% 2-mercaptoehanol and 10% FBS. 

### 2.2. Synthesis of p(NIPAAm-co-NHMAAm-co-SCC) Nanogels

Nanogels were synthesized via two-step emulsion polymerization, modified from a previous protocol [19]. Briefly, 90.4 mg of NIPAAm (80 mmole), 37.3 mg of NHMAAm (19 mmole), 0.36 mg SCC (0.05 mmole), 10 mg of MBAA (0.06 mmole) and 5.7 mg of SDS (0.02 mmole) were dissolved in 28 mL of deionized water, and then purged with nitrogen for 40 min; 10 mg of ACVA (0.04 mmole) in methanol (0.5 mL) was added to the solution and polymerization was initiated at 75 °C. After 1 h, 6.48 mg SCC (0.95 mmole) was added and stirred overnight. The product was filtered through 0.45-μm and 0.22-μm syringe filters, and then collected by centrifugation at 14,000 rpm for 40 min. The precipitate was rinsed with deionized water, and then dialyzed against deionized water (molecular weight cutoff: 12-14 KDa). The product was lyophilized and then stored at 4 °C until use.

### 2.3. Characterization of Nanogels

The size distribution of nanogels was characterized by a ZetasizerNanoZS dynamic light scattering (DLS) instrument (Malvern, UK) at a scattering angle of 173° and absorbance at 633 nm. The morphology of nanogels was imaged by transmission electron microscopy (TEM; H-7650, Hitachi, Japan). 8 μL of nanogel solution (0.4 mg/mL) was cast on to copper grids, and then negatively stained with 1% phosphotungstic acid. 

The concentration of SCC in p(NIPAAm-*co*-NHMAAm-*co*-SCC) nanogels was quantified by the absorbance at 405 nm (Cary 100 ultraviolet (UV)-visible spectrophotometer, Agilent, Santa Clara, CA, USA). The contents of SCC in the nanogels were determined from a calibration curve generated from the absorbance of a series of SCC solutions with known concentrations.

The photothermal properties of the nanogels were tested by illumination of nanogel suspension with a green laser (532 nm, Changchun New Industries Optoelectronics Tech, Changchun, China) at an intensity of 2 W/cm^2^. The temperature changes were measured by a thermocouple. Briefly, a thermocouple, 1 mm in diameter, was inserted into the nanogel suspension before and immediately after green laser irradiation.

### 2.4. Cytotoxicity of Nanogels

L929 cells were seeded in 96-well tissue culture polystyrene (TCPS) plates at a density of 2 × 10^4^ cells/cm^2^ and incubated at 37 °C for 24 h. The culture medium was replaced with a culture medium that was supplemented with SCC-nanogels at various concentrations. After 24 h of incubation, cytotoxicity of the nanogels was evaluated by an MTT assay [20]. The medium containing 0.2% phenol was used as a positive control. 100 μL of the culture medium containing MTT (5 mg MTT/mL) was added onto each sample. After incubation for 4 h, the MTT solution was discarded and 100 μL dimethyl sulfoxide (DMSO) was added for the extraction of the formed formazan. The optical absorbance of the DMSO extracts was measured at 570 nm (OD_570_) and 630 nm (OD_630_).

### 2.5. Drug Loading and Release

40 mg of dried nanogels was immersed in 10 mL of 0.4 mg/mL 5-FU in phosphate-buffered saline (PBS) solution for 20-min sonication, and then incubated at room temperature for 1 day. The drug-incorporated nanogels were precipitated by centrifugation at 13,500 rpm for 20 min and then the precipitate was rinsed with PBS. The amount of unloaded 5-FU was determined from the absorbance at 265 nm of the supernatant. The drug-loading efficiency was calculated according to the following equation:

$$\;\mathrm{Encapsulation}\;\mathrm{efficiency}\;\left(\%\right)=\;\frac{\left(\mathrm{amount}\;\mathrm{of}\;\mathrm{drug}\;\mathrm{fed}-\mathrm{amount}\;\mathrm{of}\;\mathrm{unloaded}\;\mathrm{drug}\right)}{\mathrm{amount}\;\mathrm{of}\;\mathrm{drug}\;\mathrm{fed}}\;\times 100\%\;$$

For the evaluation of drug release, the 5-FU-loaded nanogels were placed in tubes of which the ends were capped with dialysis membranes (molecular weight cutoff: 3500 Da). The tubes were placed in PBS at the temperature of either 37 or 50 °C. At every incubation interval, 1 mL PBS was sampled for the measurement of the absorbance at 265 nm in order to determine the amount of 5-FU released; 1 mL fresh PBS was replenished in each tube for continuous incubation.

In the investigation of the thermo-triggered drug release, the 5-FU-loaded nanogel suspended in PBS were incubated at 37 °C initially and then the incubation temperature was raised to 50 °C. The amount of released drug was determined. 

To determine the light-triggered drug release, the 5-FU-loaded nanogels were placed at 37 °C for 2 h and then exposed to 532-nm green laser (2 W/cm^2^) for 2 min. The amount of released drug was then determined.

### 2.6. Green Laser-Induced Cell Death in the Presence of the Nanogels

Glass coverslips (1 cm^2^) were placed in 24-well plates and sterilized by 70% ethanol, followed by overnight UV exposure. L929 cells were seeded on each glass coverslip at a density of 2 × 10^4^ cells/ cm^2^ and then incubated at 37 °C for 24 h. 500 μL of nanogel suspension in PBS was added to each well and then exposure to 532-nm green laser, followed by 2-h incubation. The cell viability was then determined by trypan blue staining. The viable and dead cells were counted under a microscope.

### 2.7. Statistical Analysis

The data were reported as mean ± standard deviation (SD). The statistical analyses between different groups were determined using Student-Keuls Multiple Comparisons Test (Instst 3.0, GraphPad Software, San Diego, CA, USA).

## 3. Results

### 3.1. Characteristics of Poly(NIPAAm-co-NHMAAM-co-SCC) Nanogels

**Figure 1:**
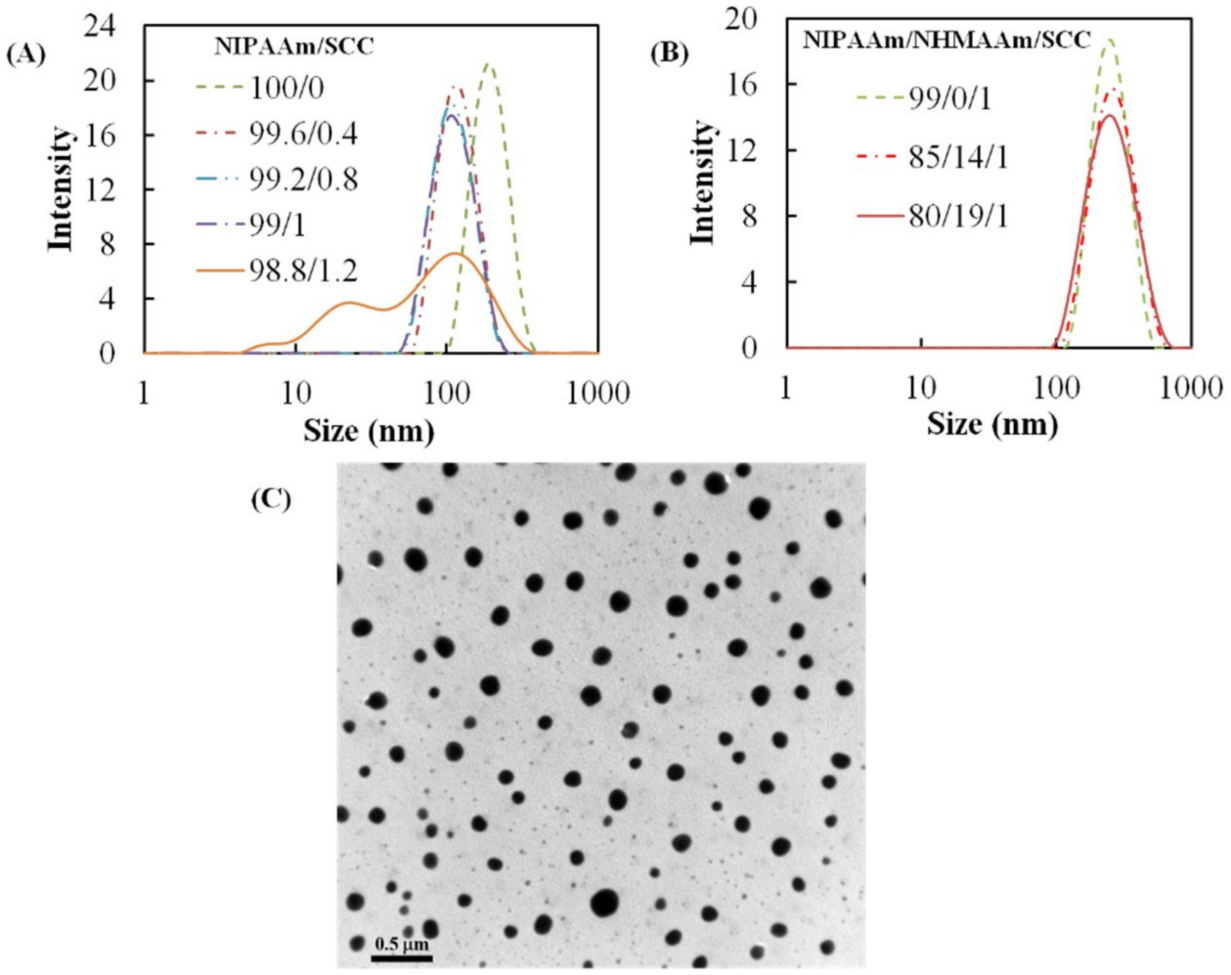
(**A**) The size distributions of the *N*-isopropylacrylamide/sodium copper chlorophyllin (NIPAAm/SCC) nanogels with various SCC concentrations; (**B**) the size distributions of NIPAAm/NHMAAm/SCC nanogels containing 1% SCC with different NIPAAm/*N*-(hydroxymethyl)acrylamide (NHMAAm) ratios; (**C**) a transmission electron microscope (TEM) image of p(NIPAAm-*co*-NHMAAm-*co*-SCC) nanogels (80/19/1) counterstained with phosphotungstic acid (average size: 133 ± 30 nm, *n* = 120).

A series of nanogels with different NIPAAm/SCC ratios was synthesized, and the sizes of the nanogels in deionized water were characterized at 25 °C using DLS. The average size of 100% pNIPAAm nanogel was ~186 nm. The addition of 1 wt % SCC decreased their average size to 102 nm (Figure 1A). When the SCC concentration was further increased to 1.2 wt %, the size distribution of the nanogel became broad and multimodal. Therefore, we decided to limit the SCC ratio to 1 wt % in the subsequent study. The addition of NHMAAm into the monomer solution did not change the average size of the nanogels (~235 nm) in spite of broader size distribution (Figure 1B). The TEM image shows that the diameters of nanogel (NIPAAm/NHMAAm/SCC= 80/19/1) ranged from 100 to 190 nm (133 ± 30 nm, Figure 1C). The amount of SCC in the nanogels (NIPAAm/NHMAAm/SCC= 80/19/1) was quantified as 0.9 wt % according to the adsorption at 405 nm, close to the feed ratio, 1 wt %.

**Figure 2:**
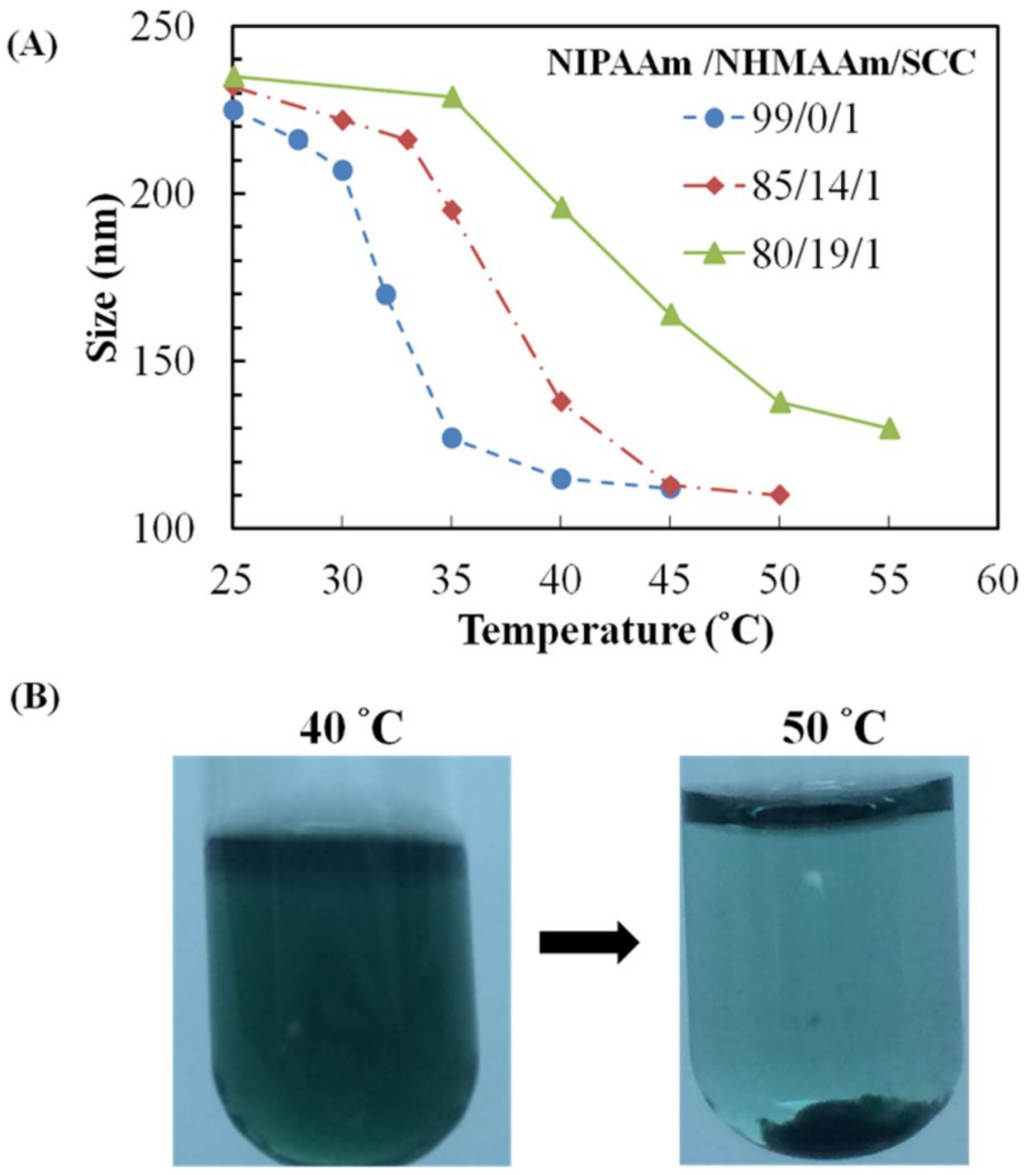
(**A**) The average sizes of the nanogels with different compositions at temperatures from 25 to 55 °C; (**B**) images for the nanogels of NIPAAm/HMAAm/SCC (80/19/1) in phosphate-buffered saline (PBS) during the temperature change from 40 to 50 °C.

The thermo-responsiveness of nanogel sizes was evaluated using DLS (Figure 2A). As expected, the nanogels was shrunk by elevated temperatures. At 40 °C, the size shrinkages of the nanogels with NIPAAm/NHMAAm molar ratio of 99/0 or 85/14 (1% SCC) are estimated to be 48% and 41% of those at 25 °C, respectively, while only 16% shrinkage appeared in the nanogels with NIPAAm/NHMAAm molar ratio of 80/19 (Figure 2A). The shrinkage is more significant with higher PNIPAAm contents. The transition temperatures of the nanogels depend on the NIPAAm/NHMAAm compositions. The temperatures at which the nanogels were shrunk by 50% were ~35, ~37 and ~42 °C for 99/0, 85/14 and 80/19 molar ratios of NIPAAm/NHMAAm, respectively (Figure 2A). As shown in Figure 2B, the nanogels (NIPAAm/NHMAAm: 80/19) were completely dispersed at 40 °C, but precipitated at 50 °C. Our results demonstrate that the addition of hydrophilic NHMAAm monomer increases the transition temperature of the copolymers. A nanogel whose transition temperature above the normal body temperature remains at a hydrated state at the normal body temperature shrinks at elevated temperatures. We expect that the nanogel (NIPAAm/NHMAAm: 80/19) could be used as a drug carrier that releases drug at a temperature above the body temperature by photothermal stimulation, so the nanogels was used in the subsequent experiments.

**Figure 3:**
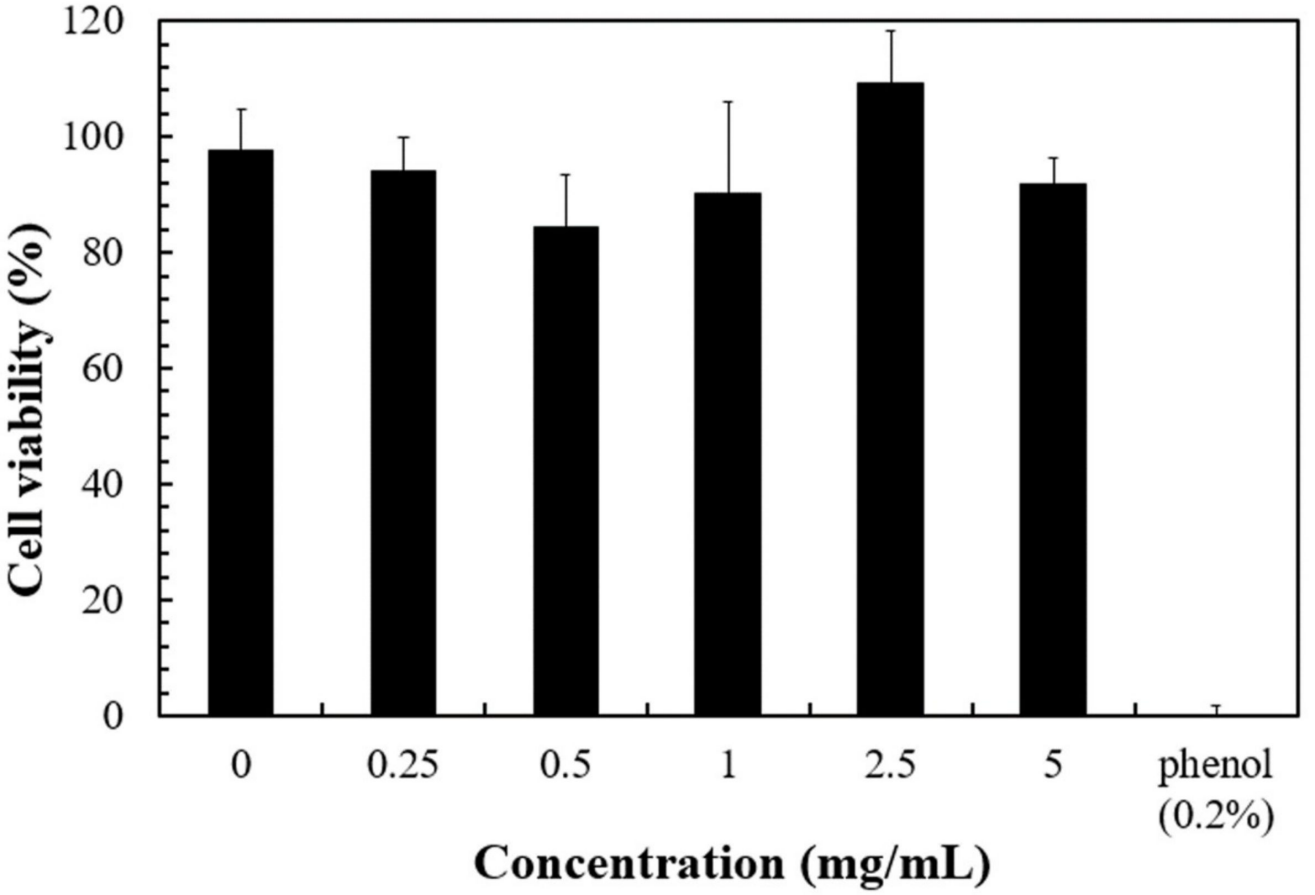
The cell viability after 1-day culture with different amount of nanogel of NIPAAm/NHMAAm/SCC (80/19/1) (*n* = 5, error bars = standard deviation (SD)).

The cytocompatibility of the nanogels is an important aspect for their clinical applications. The cytotoxicity of the nanogels was not significant even at 5 mg/mL (Figure 3). The results suggest that the nanogel should be relative safe under 5 mg/mL. Therefore, in the subsequent photothermal experiments, nanogel concentration was kept below the value.

### 3.2. Photothermal Property of the Nanogels

**Figure 4:**
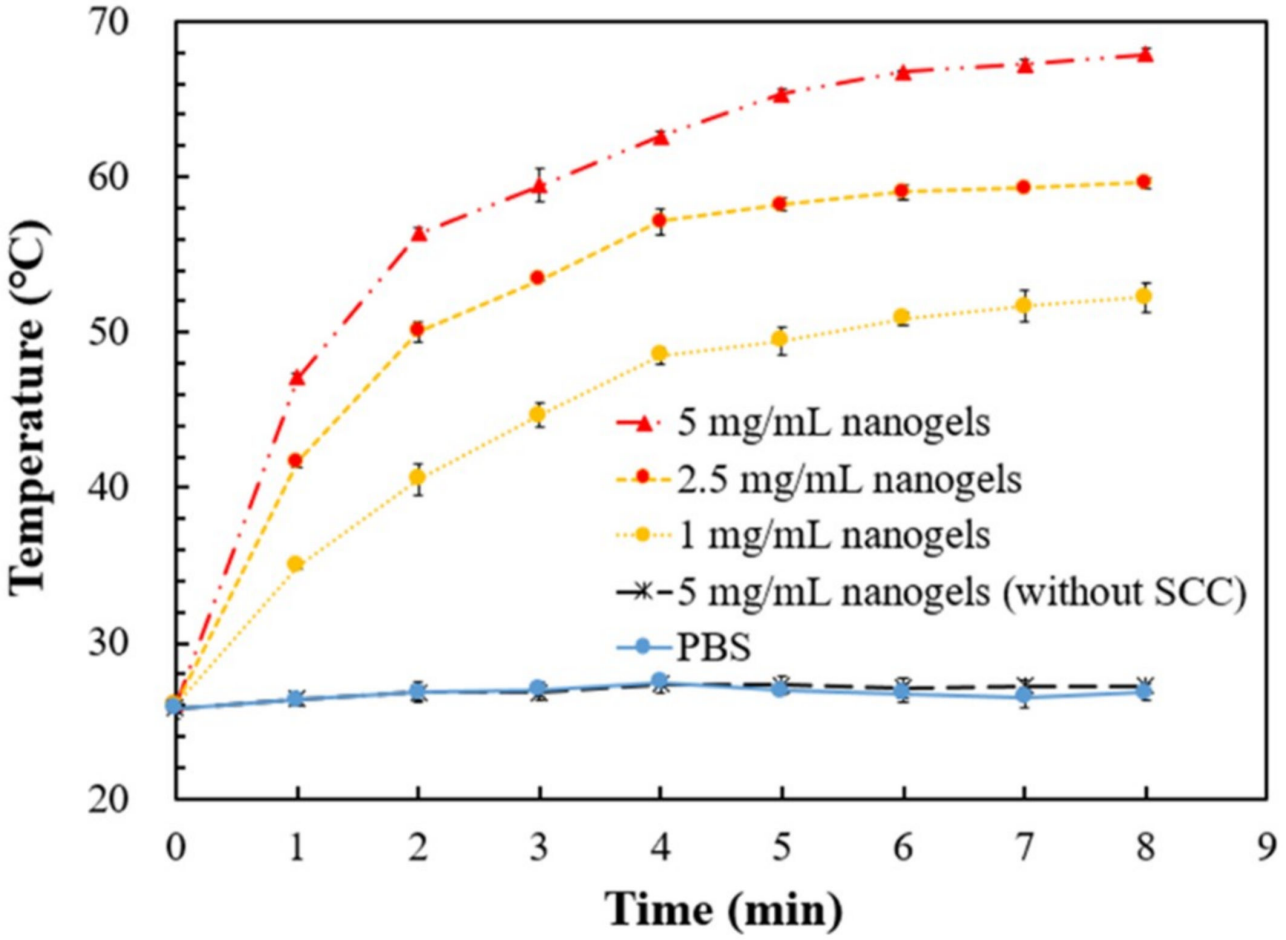
The heating curves of the solutions containing various amounts of the pNIPAAm/NHMAAm/SCC nanogel (80/19/1) after irradiation with 2 W/cm^2^ green laser (*n* = 3, error bars = SD).

The photo-induced thermal responses of the nanogels being exposed to green laser (2 W/cm^2^) were determined (Figure 4). The irradiation of green laser to PBS, or PBS containing SCC-free nanogel, did not raise temperatures after 8-min irradiation. On the other hand, the temperature of the PBS containing the nanogel (NIPAAm/NHMAAm/SCC= 80/19/1) was greatly elevated via the green-laser irradiation. The temperature increase reached a plateau of 52, 60 and 68 °C in 1, 2.5 and 5 mg/mL nanogel suspension, respectively, after 7-min exposure. The results indicate that p(NIPAAm-*co*-NHMAAm-*co*-SCC) nanogels possess a photothermal property. The temperature of the PBS containing 5 mg/mL nanogel could be raised from the room temperature to ~47 °C in merely one minute of green laser illumination. 

### 3.3. Green Laser-Induced Cell Death via the Photo-Thermal Effect of the Nanogels

**Figure 5:**
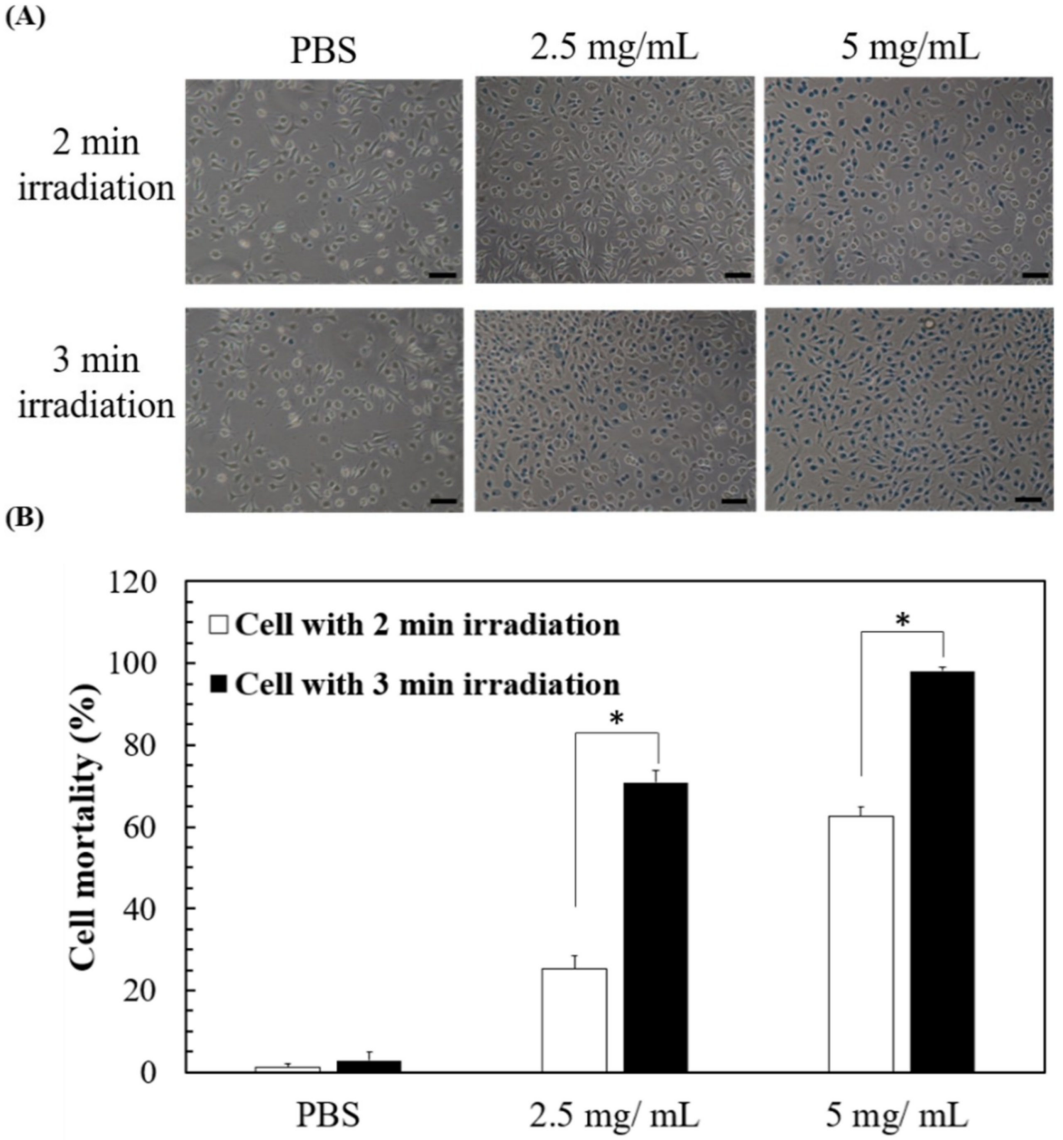
Laser-induced cell death by 2 or 3-min green laser irradiation to pNIPAAm/NHMAAm/SCC nanogel (80/19/1) at 2.5 or 5 mg/mL. (**A**) Cell morphology after green laser irradiation. Scale bar = 100 μm. (**B**) Quantitative data of cell mortality under different conditions (*n* = 3, error bars = SD). * represents *p* < 0.001.

The elevation in the temperature of the solution containing SCC-nanogel via green laser exposure could kill cells, so we next evaluated the death of L929 cells in the presence of 2.5 or 5 mg nanogel/mL under green laser exposure. The L929 cells were exposed to 2 W/cm^2^ green laser for 2 or 3 min and the cell death was then evaluated via trypan blue-exclusive staining. The trypan blue-stained images indicate that no obvious cell death was found in the absence of the nanogels, while the cells died increasingly in the presence of the nanogel (Figure 5A). The images show that longer green laser exposure causes higher cell mortality. The quantified data show that after green laser treatment, most of the experimental conditions resulted in more than 50% cell mortality (63%, 71%, and 98% for 5 mg/mL + 2-min exposure, 2.5 mg/mL + 3-min exposure, and 5 mg/mL + 3 min exposure, respectively), except in the group treated with 2.5 mg/mL nanogel + 2-min exposure (25%) (Figure 5B). The results demonstrate green laser-induced cell death by the heat generated from the nanogels even without drug loading. 

### 3.4. Drug Loading to and Release from the Nanogel

**Figure 6:**
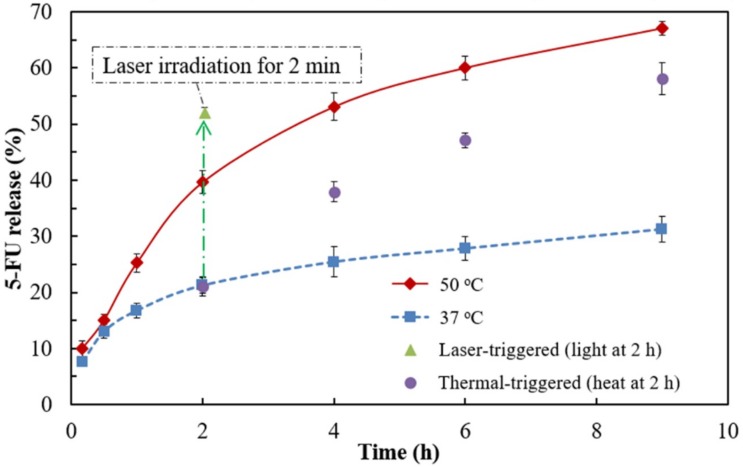
The time profile of the drug release from pNIPAAm/NHMAAm/SCC nanogel (80/19/1) in PBS at 37 or 50 °C. The thermal-trigger means nanogel-containing tubes were incubated at 37 °C for 2 h and then moved to a 50 °C incubator. On the other hand, the laser- trigger indicates the tubes were placed at 37 °C for 2 h and then exposed to the green laser (2 W/cm^2^) for 2 min. *n* = 3, error bars = standard deviation.

5-FU was chosen as a model anticancer drug for the encapsulation in the nanogel. The encapsulation capacity for 5-FU of the nanogel was 22 μg per mg nanogel. The passive drug release at 37 °C was ~21% after 2 h incubation and reached a plateau of ~31% after 9 h incubation (Figure 6A). On the other hand, the passive drug release at 50 °C was greatly enhanced to ~40% and ~67% after 2 and 9 h incubation, respectively. When the drug-loading nanogels was incubated at 37 °C for 2 h and then moved to a 50 °C environment, the extent of the drug release was increased (indicated by the purple circles in Figure 6), compared to that which remained at 37 °C. The result suggest that the thermo-responsive shrinkage of pNIPAAm-based nanogel facilitates drug release at high temperature. When the nanogels were irradiated with 2 W/cm^2^ green laser for 2 min after 2-h incubation at 37 °C, the drug release was increased suddenly from ~21% to ~52% (indicated by the green triangle in Figure 6), indicating that the drug in the nanogels could be released by a photothermal effect.

### 3.5. The Cell-Killing Efficacy of 5-FU-Loaded Nanogels under Green Laser Irradiation

**Figure 7:**
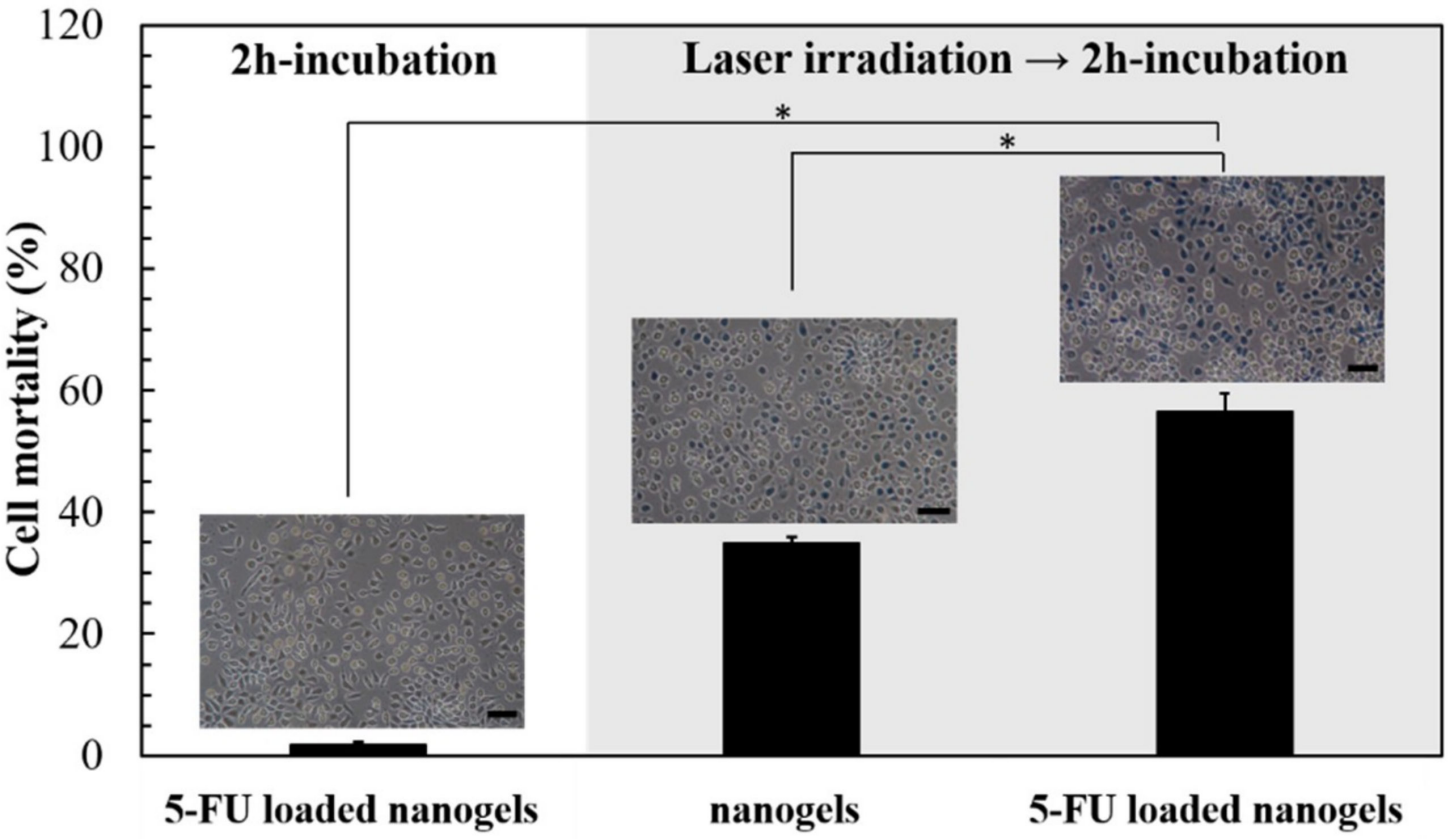
Laser-induced cell death owing to 5-FU loaded pNIPAAm/NHMAAm/SCC nanogel (80/19/1) (*n* = 3, error bars = SD). After 2 h of incubation, cell mortality was evaluated by trypan blue staining. Scale bar = 100 μm. * represents *p* < 0.001.

After L929 cells were incubated with the nanogels containing 55 μg 5-FU for 2 h, the cell death was not significant (1.7%, Figure 7). When L929 cells were incubated with the 5-FU-free nanogel and irradiated by green laser for 2 min, the cell mortality was increased to 35%. When the L929 cells were incubated with the 5-FU-loaded nanogel and irradiated by green laser for 2 min, the cell mortality was further increased to 56%. The results indicated the cell death could be enhanced by the synergetic effect of the anti-cancer 5-FU and photothermal heat.

## 4. Discussion

p(NIPAAm-*co*-NHMAAm-*co*-SCC) nanogels were prepared by a two-step surfactant free emulsion polymerization. At the beginning, we experienced difficulty in the preparation of SCC-incorporated nanogels. After several trial-and-error attempts, we found that a two-step addition of SCC, a small initial amount (0.05 mmole) followed by a large amount (0.095 mmole), is needed for stable synthesis of p(NIPAAm-*co*-NHMAAm-*co*-SCC) nanogel (see Section 2.2). This two-step emulsion polymerization has been reported for the preparation of the poly(styrene-*co*-*N*-isopropylacrylamide) core-shell microspheres [19]. The first small SCC addition seems to stabilize the precursor nanogel, thus facilitating the growth of the nanogel during the second addition of a larger amount of SCC. It should be noted that, to prevent the aggregation nanogel, a small amount of the surfactant, SDS whose concentration is far less than critical micelle concentration, was used during the polymerization. The sizes of the nanogels fabricated in this study is smaller in comparison to the pNIPAAm-based nanogels produced via similar surfactant-free emulsion polymerization [21,22]. The results indicate that the incorporation of SCC may decrease the sizes of nanogels. We conjecture that the porphyrin structure of SCC may hinder the polymerization sterically. Furthermore, when the amount of SCC exceeds a certain range (e.g., 1.2%), the nanogel sizes display a multimodal distribution (Figure 1A). It is known that porphyrin-type molecules form π–π stacking, which may increase the complexity of polymerization [23].

We previously showed that SCC-containing nanogels based on *N*-[3-(dimethylamino) propyl] methacrylamide (NDPMA) possess a photothermal capacity and could be used for photo-induced hyperthermia therapy [17]. However, the cancer-killing capacity of SCC-containing nanogel could be further improved. In order to enhance the anti-cancer capacity, we added a temperature-responsive property to SCC-containing nanogels for the control of the release of anti-cancer agents. The SCC-containing pNIPAAm-based nanogels fabricated in this study still possess a photo-induced hyperthermia efficacy to kill cells (Figure 5). Furthermore, the nanogel could be loaded with anti-cancer drugs, e.g., 5-FU, and could release the drug when the nanogel is heated via green laser illumination. Our results indicate that the drug is released promptly owing to the laser-trigger (Figure 6B), which should be ascribed to rapid shrinkage of the nanogel at an abrupt temperature raise. The drug release behavior is similar to the observation by Guo et al. [24]. In their study, the photothermal effect was induced by UV light, which may damage the cells. Furthermore, our nanogel releases the drug at a temperature higher than the normal LCST of pNIPAAm, leading to the nanogels exhibiting a good potential for in vivo application.

L929 cell was incubated with the 5-FU loaded nanogels and exposed to green laser. The results reveal that, compared to the nanogels without irradiation or without 5-FU loading, 5-FU loaded nanogels exert an additive cyto-killing effect at green laser illumination (Figure 7). It has been reported that the synergistic combination of hyperthermia and 5-FU release would promote cellular apoptosis, suggesting that heat might change the permeability of cell membrane, leading to liability for drug penetration into cells [25]. Therefore, our results suggest that heat generated by SCC might cause the thermo-chemotherapy effect, enhancing cell mortality.

Although the nanogels prepared in this study possess an enhanced cyto-killing property based on photothermia and drug release, there is still room for further improvement. First, the encapsulation efficiency and drug-loading capacity of nanogels could be increased, which might be achieved by the formation of polymer-drug conjugates via reversible disulfide [26], ester [27], or hydrazone [28,29] linkages. Second, the cellular uptake efficiency of nanogels could be improved by conjugation of ligands to surface receptors of cancer cells, such as peptides [30] or galactose [31], leading to specific cell recognition and active endocytosis. With further improvement, the p(NIPAAm-*co*-NHMAAm-*co*-SCC) nanogels should benefit photothermal cancer therapy.

## 5. Conclusions

In this study, p(NIPAAm-*co*-NHMAAm-*co*-SCC) nanogel with low cytotoxicity has been successfully fabricated via two-step emulsion polymerization. The photothermal and thermoresponsive properties of the nanogel were modulated by the amount of SCC and NHMAAm. Anticancer drugs could be loaded in the nanogels and be promptly released upon exposure to green laser light. The syngeneic effect of the anti-cancer drug and photo-induced hyperthermia is demonstrated by the enhanced cell mortality. The photothermo-responsive nanogel possesses a great potential in anti-cancer treatment.
